# Supplementary material for: Decline in HIV prevalence among female sex workers in Zimbabwe between 2013 and 2023
Source: Nat Commun. 2025 Dec 5;16:10912. doi: 10.1038/s41467-025-65901-x (PMC12680614; doi:10.1038/s41467-025-65901-x)
Supplement: Supplementary file 2 — Reporting Summary [file 41467_2025_65901_MOESM2_ESM.pdf]

nature portfolio

Corresponding author(s): DBPR NCOMMS-25-03675A

Last updated by author(s): Jul 10, 2025

Reporting Summary

Nature Portfolio wishes to improve the reproducibility of the work that we publish. This form provides structure for consistency and transparency in reporting. For further information on Nature Portfolio policies, see our Editorial Policies and the Editorial Policy Checklist.

Statistics

For all statistical analyses, confirm that the following items are present in the figure legend, table legend, main text, or Methods section.

n/a	Confirmed	
☐	☒	The exact sample size (*n*) for each experimental group/condition, given as a discrete number and unit of measurement
☐	☒	A statement on whether measurements were taken from distinct samples or whether the same sample was measured repeatedly
☐	☒	The statistical test(s) used AND whether they are one- or two-sided<br>*Only common tests should be described solely by name; describe more complex techniques in the Methods section.*
☐	☒	A description of all covariates tested
☒	☐	A description of any assumptions or corrections, such as tests of normality and adjustment for multiple comparisons
☐	☒	A full description of the statistical parameters including central tendency (e.g. means) or other basic estimates (e.g. regression coefficient) AND variation (e.g. standard deviation) or associated estimates of uncertainty (e.g. confidence intervals)
☒	☐	For null hypothesis testing, the test statistic (e.g. *F*, *t*, *r*) with confidence intervals, effect sizes, degrees of freedom and *P* value noted<br>*Give P values as exact values whenever suitable.*
☒	☐	For Bayesian analysis, information on the choice of priors and Markov chain Monte Carlo settings
☐	☒	For hierarchical and complex designs, identification of the appropriate level for tests and full reporting of outcomes
☒	☐	Estimates of effect sizes (e.g. Cohen's *d*, Pearson's *r*), indicating how they were calculated

Our web collection on statistics for biologists contains articles on many of the points above.

Software and code

Policy information about availability of computer code

Data collection	We did not directly collect data from participants but original data was collected using ODK (Open Data Kit) on Android Tablets.
Data analysis	The code used to analyse the data will be publicly available on an online repository. Analyses were performed using R programming language (version 4.4.2).

For manuscripts utilizing custom algorithms or software that are central to the research but not yet described in published literature, software must be made available to editors and reviewers. We strongly encourage code deposition in a community repository (e.g. GitHub). See the Nature Portfolio guidelines for submitting code & software for further information.

Data

Policy information about availability of data

All manuscripts must include a data availability statement. This statement should provide the following information, where applicable:

- Accession codes, unique identifiers, or web links for publicly available datasets
- A description of any restrictions on data availability
- For clinical datasets or third party data, please ensure that the statement adheres to our policy

We used data collected from other studies using respondent-driven sampling surveys conducted among cisgender female sex workers aged 18 and above in 2013, 2016, and 2017 across thirteen towns, and in 2021 and 2023 across two cities. We pooled all the RDS surveys from these time-points to create an analytic dataset, which will be available on an online repository.

Research involving human participants, their data, or biological material

Policy information about studies with human participants or human data. See also policy information about sex, gender (identity/presentation), and sexual orientation and race, ethnicity and racism.

Reporting on sex and gender	Original data collection was restricted to cisgender female sex workers.
Reporting on race, ethnicity, or other socially relevant groupings	We did not include socially constructed groupings in our analysis, although data on participant tribe was collected in the original studies.
Population characteristics	We analysed characteristics that were collected in the original studies which include age, socio-demographics, sex work, sexual behaviour, HIV prevention, HIV care uptake, and personal network size for each participant for RDS adjustment.
Recruitment	We did not recruit participants for this particular study. We used data we collected from previous studies (i.e., secondary data analysis).
Ethics oversight	The study does not require ethical approval because we analysed data that was previously collected for other purposes. However, the original studies were approved by the Medical Research Council of Zimbabwe (MRCZ) and the Research Council of Zimbabwe (RCZ), as well as ethics committees for collaborator institutions, including the Liverpool School of Tropical Medicine, University College London, and the London School of Hygiene and Tropical Medicine.

Note that full information on the approval of the study protocol must also be provided in the manuscript.

Field-specific reporting

Please select the one below that is the best fit for your research. If you are not sure, read the appropriate sections before making your selection.

☐ Life sciences ☒ Behavioural & social sciences ☐ Ecological, evolutionary & environmental sciences

For a reference copy of the document with all sections, see nature.com/documents/nr-reporting-summary-flat.pdf

Behavioural & social sciences study design

All studies must disclose on these points even when the disclosure is negative.

Study description	We used quantitative cross-sectional data from five separate multi-site respondent-driven sampling surveys conducted in 2013, 2016, 2017, 2021 and 2023.
Research sample	We restricted our analysis to a research sample of cisgender female sex workers aged 18-39 from fifteen urban areas in Zimbabwe. The data sources are five separate multi-site respondent-driven sampling surveys conducted among cisgender female sex workers aged 18 and above in 27 rural, peri-urban and urban areas in Zimbabwe.
Sampling strategy	We aimed to utilise the existing serial respondent-driven sampling surveys data for locations with repeat surveys collected among cisgender female sex workers in Zimbabwe, so we could analyse changes over time in HIV prevalence. Therefore, we did not conduct an a priori power calculation to determine a suitable sample size linked to a particular level of significance. Instead, the sample size was the number of cisgender female sex workers who participated in five respondent-driven sampling surveys in 2013, 2016, 2017, 2021 and 2023 across 27 rural, peri-urban, and urban areas in Zimbabwe.
Data collection	In this study, we did not directly collect data from participants. Instead, we analysed data from existing datasets after pooling the original datasets across time-points to create an analytic dataset. The original data was collected using computer-assisted personal interviewing (CAPI) in 2013, 2016, and 2017, and audio computer-assisted self-interviewing (ACASI) in 2021 and 2023.
Timing	We included cross-sectional data that was collected in 2013, 2016, 2017, 2021, and 2023.
Data exclusions	We conducted five separate multi-site respondent-driven sampling surveys across 27 rural, peri-urban, and urban areas. For our analysis, we excluded data from 12 of these locations (N=2419) because they lacked repeat surveys. We also excluded data from female sex workers aged 40 and above (n=2508). Our focus was on locations with repeat surveys, enabled us to analyse changes in HIV prevalence over time. We focused on female sex workers under 40 because HIV prevalence changes in this age group are more likely to reflect HIV incidence patterns. Additionally, data for older female sex workers were sparse.
Non-participation	In this study, we can't document non-participation as we did not directly collect data from participants – we analysed data from existing datasets (i.e., secondary data analysis).
Randomization	Participants were not allocated to experimental groups.

Reporting for specific materials, systems and methods

We require information from authors about some types of materials, experimental systems and methods used in many studies. Here, indicate whether each material, system or method listed is relevant to your study. If you are not sure if a list item applies to your research, read the appropriate section before selecting a response.

Materials & experimental systems

n/a	Involved in the study
☒	☐ Antibodies
☒	☐ Eukaryotic cell lines
☒	☐ Palaeontology and archaeology
☒	☐ Animals and other organisms
☒	☐ Clinical data
☒	☐ Dual use research of concern
☒	☐ Plants

Methods

n/a	Involved in the study
☒	☐ ChIP-seq
☒	☐ Flow cytometry
☒	☐ MRI-based neuroimaging

Plants

Seed stocks	*Report on the source of all seed stocks or other plant material used. If applicable, state the seed stock centre and catalogue number. If plant specimens were collected from the field, describe the collection location, date and sampling procedures.*
Novel plant genotypes	*Describe the methods by which all novel plant genotypes were produced. This includes those generated by transgenic approaches, gene editing, chemical/radiation-based mutagenesis and hybridization. For transgenic lines, describe the transformation method, the number of independent lines analyzed and the generation upon which experiments were performed. For gene-edited lines, describe the editor used, the endogenous sequence targeted for editing, the targeting guide RNA sequence (if applicable) and how the editor was applied.*
Authentication	*Describe any authentication procedures for each seed stock used or novel genotype generated. Describe any experiments used to assess the effect of a mutation and, where applicable, how potential secondary effects (e.g. second site T-DNA insertions, mosiacism, off-target gene editing) were examined.*
